# Prevalence of anemia among under-5 children in the Ghanaian population: estimates from the Ghana demographic and health survey

**DOI:** 10.1186/1471-2458-14-626

**Published:** 2014-06-19

**Authors:** Joycelyne E Ewusie, Clement Ahiadeke, Joseph Beyene, Jemila S Hamid

**Affiliations:** 1 Department of Mathematics and Statistics, McMaster University, Hamilton, Canada; 2 Institute of Statistical, Social and Economic Research, University of Ghana, Legon, Ghana; 3 Department of Clinical Epidemiology and Biostatistics, McMaster University, 1280 Main Street West, Hamilton, Ontario L8S 4 K1, Canada; 4 Department of Pathology and Molecular Medicine, McMaster University, Hamilton, Canada

**Keywords:** Anemia, Prevalence, Micronutrient deficiency, Children, Ghana, Ghana demographic and health survey

## Abstract

**Background:**

Anemia in children continues to be a major public health challenge in most developing countries, particularly in Africa. Anemia in the early stages of life leads to severe negative consequences on the cognitive as well as the growth and development of children, which may persist even after treatment. We examine the prevalence of anemia in under-five children in the Ghanaian population to help inform and serve as a guide to health policies and possible interventions.

**Methods:**

Data from the 2008 Ghana Demographic and Health Survey (GDHS) was used. Data consists of health, demographic and socio-economic factors. Anemia status was determined using hemoglobin level, and prevalence of childhood anemia along with 95% confidence intervals was provided. We also examined the distribution of prevalence across different age and socio-demographic groups as well as the different regions and sub-regions in Ghana.

**Results:**

The overall prevalence of anemia in under-five children in Ghana was 78.4% (N = 2168, 95% CI: 76.7-80.2), where 7.8% (N = 2168, 95% CI: 6.6-8.9) of the children had severe anemia, 48.0% (N = 2168, 95% CI: 45.9-50.2) moderate anemia and 22.6% (N = 2168, 95% CI: 20.8-24.4) had mild anemia. The highest prevalence regions were the Upper East, 88.9% (N = 158, 95% CI: 80.9-94.0), and Upper West 88.1% (N = 220, 95% CI: 76.4-94.6). The prevalence was also higher among children under 2 years of age, 85.1% (N = 781, 95% CI: 82.6-87.7) than children 2–5 years of age, 74.8% (N = 1387, 95% CI: 72.5-77.1). No significant difference in prevalence between boys and girls was observed.

**Conclusions:**

Given the high prevalence of childhood anemia observed in Ghana, particularly among those less than 2 years old, and given the negative consequences on their cognitive and behavioral development even in later years, there is an urgent need for effective and efficient public health interventions.

## Background

Anemia is one of the most serious public health problems affecting people in both developing and industrialized countries. In developing countries, it is reported that an estimated 3.5 billion people are anemic [1]. The 2008 World Health Organization (WHO) estimate of anemia prevalence in Africa was 64.6%, which is almost 50 percentage points higher than the prevalence in Europe (16.4%), and over 60 percentage points higher than in North America (3.4%) [2]. Anemia in children is of particular interest since it impairs their mental, physical and social development; it causes negative behavioral and cognitive effects resulting in poor school performance and work capacity in later years [3]. Iron deficiency is indicated as the most common cause of anemia in under-five children with a smaller proportion due to other micronutrient deficiency such as folate, Vitamin A and B12 [3-5].

Several studies have shown that iron deficiency anemia during the first two years of life leads to impairments in the cognitive and behavioral development that persist even after treatment of iron deficiency [6,7]. Despite the serious health and social implications, prevalence of anemia remains a major public health concern and is indicated as one of the leading causes of infant mortality and morbidity in developing countries, in particular countries across Africa [8,9]. For instance, a study of anemia in children along the coast of Tanzania reports a prevalence of 74% [10]. Another study in the Democratic Republic of Congo estimates a prevalence of 43% [11]. In a study conducted in Southern Cameroon, the prevalence in children less than 2 years was found to be 45% [4]. In other developing countries, the prevalence of childhood anemia ranges from 27.2% in Mexico to 60.6% in Haiti [3,12].

In this study, we performed a comprehensive investigation of childhood anemia in the Ghanaian population using data from the Ghana Demographic and Health Survey (GDHS). We examined the prevalence in the overall population and within some of the categories considered in the GDHS report and compared our results to the findings. We also performed detailed investigation of the distribution of anemia prevalence with respect to several age categories within the different socio-economic groups as well as within the different regions and sub-regions in Ghana.

National and regional estimates of the prevalence of anemia are provided in the 2008 GDHS report (78.4% overall; 84.1% in rural; 67.9% in urban) [13]. Thus policy makers, program planners and other non-governmental organizations are informed of the high prevalence of anemia. What is widely unknown is the prevalence within specific socio-demographic subgroups as well as sub-regional areas with specific needs. Due to this lack of information, directing the limited resources to the appropriate target areas is mostly a challenge and a contributing factor to missing the target population. Furthermore, identifying the specific variables associated with anemia is relevant in prioritizing interventions and revealing patterns for improved results.

## Methods

### The Ghana demographic and health survey

The Ghana Demographic and Health Survey (GDHS) is part of the international Demographic and Health Survey (DHS) program, led by the United States Agency for International Development (USAID), which has been conducting household surveys in developing countries since 1984 [14]. In Ghana, the DHS is conducted every five years and the first round was conducted between February and June 1988. The survey used a stratified, two-stage cluster design, where, a total of 412 clusters, which consisted of 182 clusters in urban areas and 230 in rural areas were chosen [13]. Details about the survey can be found in the 2008 GDHS report [13]. Since the objective of this research was to investigate the prevalence of anemia in children under the age of five, the Children’s Data Recode File part of GDHS data sets was used [13].

### Weighting the sample

Sample weights were applied in order to compensate for the unequal probability of selection between the strata that has been geographically defined as well as for non-response. A detailed explanation of the weighting procedure can be found in the Demographic and Health Survey Methodology report [15].

### Ethical approval

The ethical approval for the GDHS was received from the Ghana Health Service Ethical Review Committee, Accra, Ghana. No further ethical approval was needed for this study because the anonymous data, with no personal identifier or link, was received from the Ghana Statistical Service.

### Statistical analysis

Children under five years of age were investigated for hemoglobin concentration, birth weight, gender, place of residence, region, incidence of an infection, and financial status. Birth weight as reported subjectively by the mother of the child was grouped into 3 categories: large, average and small. Type of place of residence was categorized as rural, if it was a countryside and urban otherwise. The region of residence refers to the geographic region, where the respondent was interviewed, and the country is divided into 10 geographic regions. Infection indicators, such as diarrhea, fever, or cough, in the last two weeks preceding the survey, were also considered in the analysis. Financial status was grouped into 3 categories based on the GDHS wealth quintiles: high, middle, and low income families. Age was first divided into 5 subgroups and then into 2 subgroups (less than 2 years old and between 2 and 5 years old). This is similar to what is found in literature [3,4,12].

The primary outcome of interest was the prevalence of anemia. Anemia is defined as a hemoglobin level of less than the 5th percentile for age, and has several causes which vary by age [16]. According to WHO, children under 5 years of age with hemoglobin level less than 11.0 g/dl are considered anemic [2]. The cut-off values for the various levels of severity were: <7 · 0 g/dl for severe anemia, 7 · 0 g/dl-9 · 9 g/dl for moderate anemia and 10 · 0 g/dl-10 · 9 g/dl for mild anemia. Prevalence estimates, along with 95% confidence intervals (CIs), were provided for all the sub-groups considered. Statistical differences among the groups were examined using a chi-square test. Analyses were performed using the R statistical software (version 3 · 0 · 1; The R Foundation for Statistical Computing) and data organization was done using SPSS for windows (version 20; IBM SPSS Inc., 2012) [17,18].

## Results

The Children’s Recode dataset consisted of 2992 children aged 6–59 months. However, 2168 children, for whom the primary outcome (hemoglobin level) was available, were included in the analysis, of whom 1073 (49.5%) were female and 1095 (50.5%) were male. The mean age (± SD) was 31.6 ±15·7 months (2.2 ± 1.3 years). Information on birth weight was available for 2149 of the 2168 children and data on the symptoms of illness or infection (such as diarrhea and fever) was provided for 2164 children.

The most represented age group in the sample was children aged 12–23 months old, (age category 1 ≤ age< 2). This group was made up of 521 (24%) children, while those who were 6 months but had not yet celebrated their 1st birthday were the least represented, 260 (12%). Those who were 2 but not yet 3 years were 465 (21.4%) and 432 (19.9%) of them were aged 3 but not yet 4. The last group involved 490 (22.6%) children aged 4 years but not yet 5. About 695 (32%) respondents were in urban residences, while 1473 (68%) were in rural residences. There were twice as many children from low income households, 1223 (56.4%), as there were from high income households, 601 (27.7%). A total of 344 (15.9%) children were from middle income households.

The overall prevalence of anemia among children under 5 was 78.4% (N= 2168, 95% CI: 76.7-80.2), where 7.8% (N= 2168, 95% CI: 6.63-8.91) of the children had severe, 48% (N= 2168, 95% CI: 45.9-50.2) moderate and 22·6% (N= 2168, 95% CI: 20.8-24.4) had mild anemia. This indicates that over 55% of the children were at least moderately anemic (Hb level<9·9 g/dl). These results are consistent with the overall prevalence reported in the 2008 GDHS. In spite of this overall prevalence, heterogeneity in the data resulted in different prevalence estimates across most categories. Table 1 provides the distribution of anemia across the different age groups, gender and socio-economic status.

**Table 1:** Overall prevalence of anemia by age, gender, financial status and birth weight

**Characteristic**		**Sample size(n)**	**Prevalence of anemia % (Weighted)**	**Prevalence of anemia % (Unweighted)**	p- value*
			**(95% confidence interval)**	**(95% confidence interval)**	
Age** in years	**Total**	2168	78·4 (76·7 - 80·2)	80·1 (78·4 - 81·8)	< 0·0001
	6 months<age< 1	260	85·1 (80·5 - 89·6)	86·5 (82·2 - 90·6)	
	1≤ age < 2	521	85·1 (81·9 - 88·2)	87·1 (84·2 - 90·0)	
	2≤ age < 3	465	80·1 (76·2 - 83·7)	81·1 (77·5 - 84·7)	
	3≤ age <4	432	74·1 (69·8 - 78·0)	75·5 (71·2 - 79·4)	
	4≤ age <5	490	70·5 (66·2 - 74·4)	72·2 (68·1 - 76·1)	
	< 2	781	85·1 (82·6-87·7)	86·9 (84·6- 89·3)	< 0·0001
	2≤ age <5	1387	74·8 (72·5-77·1)	76·2 (73·6 – 78·5)	
**Gender**	Male	1095	79·6 (77·1 - 81·9)	81·4 (78·9 - 83·6)	0·1670
	Female	1073	77·1 (74·4 - 79·6)	78·8 (76·2 - 81·2)	
**Financial status**	Low	1223	85·6 (83·4 - 87·7)	86·2 (84·2 - 88·1)	< 0·0001
	Middle	344	81·0 (76·7 - 84·8)	81·1 (76·9 - 85·2)	
	High	601	66·3 (62·6 - 69·7)	67·1 (63·2 - 70·8)	
Birth weight***	Large	1179	77·3 (74·9 - 79·7)	79·2 (76·9 - 81·5)	0·4530
	Average	664	79·3 (76·1 - 82·3)	80·6 (77·6 - 83·6)	
	Small	306	80·1 (75·5 - 84·8)	81·7 (77·3 - 86·1)	
Has symptoms of infection***	Yes	945	83·3 (80·7 - 85·6)	84·4 (81·9 - 86·7)	< 0·0001
	No	1219	74·6 (72·0 - 77·1)	76·7 (74·2 - 79·0)	

*p-value shows the difference in prevalence across the various groups within each category.

**only children aged from 6 months to below 5 years are included in the analysis.

***the analysis did not include children with missing information on birth weight and on infection or illness indicators respectively.

The prevalence in infants less than a year old was as high as that recorded in infants aged between 1 and 2 years old at 85.1% (N=521, 95% CI: 81.9-88.2). The prevalence decreased consistently with age, reaching 70.5% (N=490, 95% CI: 66.2-74.4) in later years. Children who were less than 2 years old had a higher prevalence than older children (Table 1). The prevalence of anemia in male children was not significantly different from that of female children (p=0.167). However, for younger children less than 2 years, the prevalence among those who were still being breastfed was significantly higher, 87.3%, (N=674, 95% CI: 84.4-89.8) than those who had been weaned, 74.2% (N=107, 95% CI: 65.4-81.4). In the older categories, the difference in prevalence between breastfed and weaned children was not statistically significant (p =0.157). The prevalence for children from low income households was significantly higher, 85.6% (N=1223, 95% CI: 83.4-87.7) than children from high income households, 66.3% (N=601, 95% CI: 62.6-69.7). Although the prevalence was lower in children with large birth weight than in those with small birth weight, the difference was not statistically significant (p = 0.453). The prevalence of anemia for children with at least one symptom of infection was significantly higher (p<0.0001) than the prevalence among children with no symptoms.

The prevalence of anemia for the urban population was lower (67.5%, N=695, 95% CI: 64.3-70.9) than that of the general population (78.4%, N=2168, 95% CI: 76.7-80.2), and much lower than that of the rural population (84.8%, N=1473, 95% CI: 82.8-86.7) (Table 2). That is, prevalence of anemia among rural children is about 17 percentage points higher than that of urban children. The highest prevalence for the urban areas was recorded among children between the age of 1 and 2 (81.2%, N=176, 95% CI: 75.7-86.9). This prevalence is also lower than what is observed in the general population for the same age category (85.1%, N=521, 95% CI: 81.9-88.2). In the rural areas, the highest estimates were recorded in infants less than a year old (90.1%, N=187, 95% CI: 85.5-94.8) and those aged between 1 and 2 years, (87.5%, N=345, 95% CI: 83.6-91.0). Rural infants in these age categories had a much higher prevalence of anemia, compared to the general population (Table 2).

**Table 2:** Prevalence of anemia by age, sex and place of residence

	**Characteristic**	**Sample size (n)**	**Prevalence of anemia % (Weighted)**	**Prevalence of anemia % (Unweighted)**	p-value*
**Type of residence**			**(95% confidence interval)**	**(95% confidence interval)**	
**Urban**	**Total**	695	67·5 (64·3 – 70·9)	68·8 (65·3 – 72·2)	
	Age** (Years)				
	6 months<age< 1	73	75·0 (64·6 - 84·4)	75·3 (64·8 - 85·3)	
	1≤ age < 2	176	81·2 (75·7 - 86·9)	81·2 (75·2 - 87·0)	
	2≤ age < 3	149	69·1 (61·9 - 76·3)	69·8 (62·6 - 77·6)	< 0·0001
	3≤ age <4	150	61·6 (54·1 - 68·6)	62·7 (54·5 - 70·3)	
	4≤ age <5	147	53·2 (45·5 - 60·7)	55·8 (47·3 - 63·6)	
	< 2	398	79·4 (74·7-84·4)	79·5(74·5-84·6)	< 0·0001
	2≤ age <5	297	61·3 (57·0-65·5)	62·8 (58·3-67·3)	
	**Sex**				
	Male	350	68·2 (63·6 - 72·7)	70·0 (65·2 - 74·8)	0·694
	Female	345	66·8 (61·7 - 71·3)	67·5 (62·6 - 72·5)	
	**Total**	1473	84·8 (82·8 - 86·7)	85·4 (83·6 – 87·2)	
**Rural**	Age** (Years)				
	6 months<age< 1	187	90·1 (85·5 - 94·8)	90·9 (86·7 - 95·0)	
	1≤ age < 2	345	87·5 (83·6 - 91·0)	90·1 (86·9 - 93·3)	
	2≤ age < 3	316	86·2 (82·0 - 90·1)	86·4 (82·4 - 90·1)	0·015
	3≤ age <4	282	82·6 (77·8 - 87·1)	82·3 (77·6 - 86·6)	
	4≤ age <5	343	80·0 (75·4 - 84·4)	79·3 (74·9 - 63·6)	
	< 2	848	88·4 (85·5-91·3)	90·4 (87·9 - 92·9)	0·006
	2≤ age <5	625	82·8 (80·3-85·3)	82·6 (80·1 – 85·0)	
	**Sex**				
	Male	745	86·3 (83·5 - 88·8)	86·7 (84·3 - 89·2)	0·108
	Female	728	83·2 (80·3 - 86·1)	84·1 (81·4 - 86·7)	

*p-value shows the difference in prevalence across the various groups within each category.

**only children aged from 6 months to below 5 years are included in the analysis.

**Figure 1:**
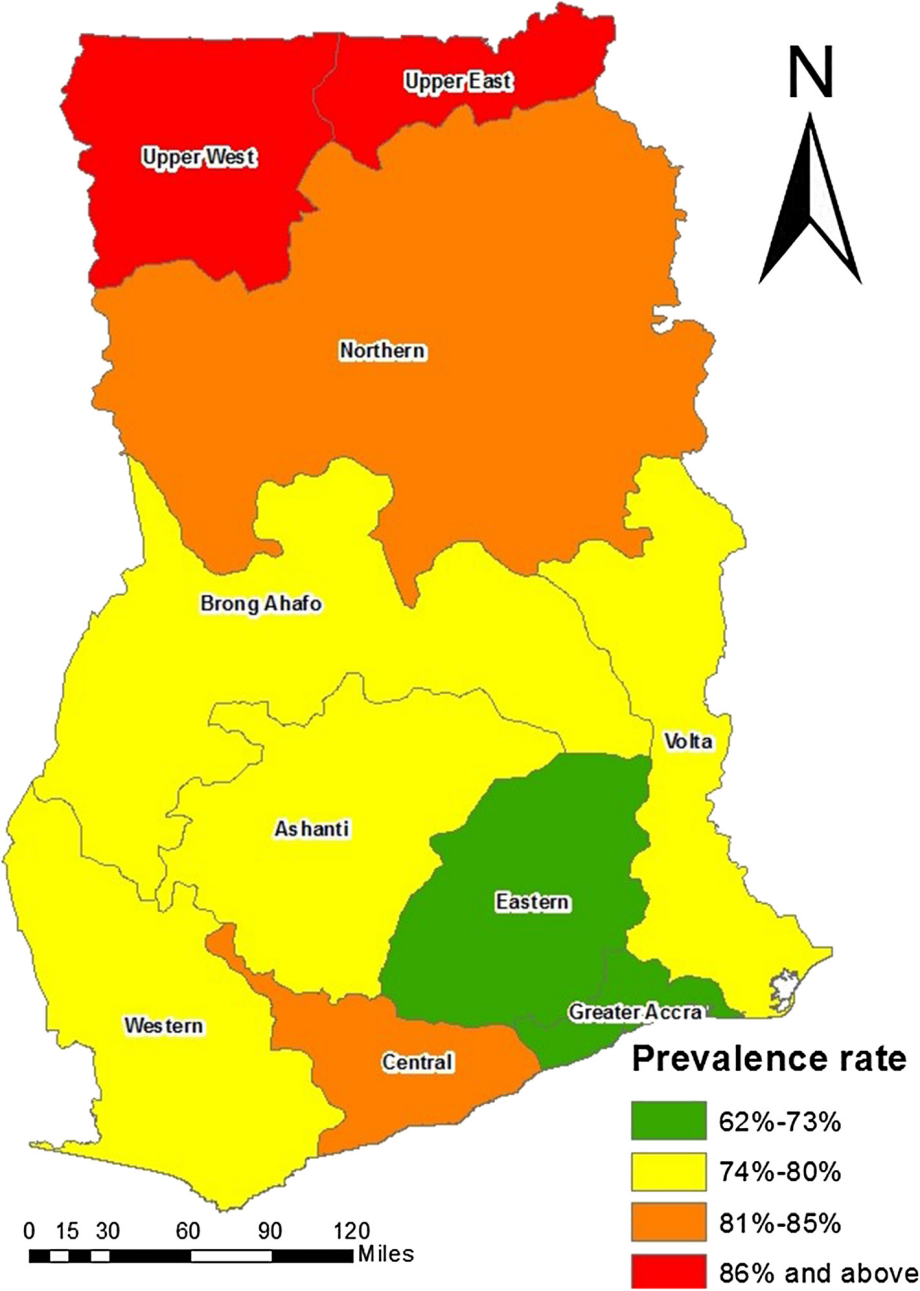
Distribution of childhood anemia by region in under-five children in Ghana.

**Figure 2:**
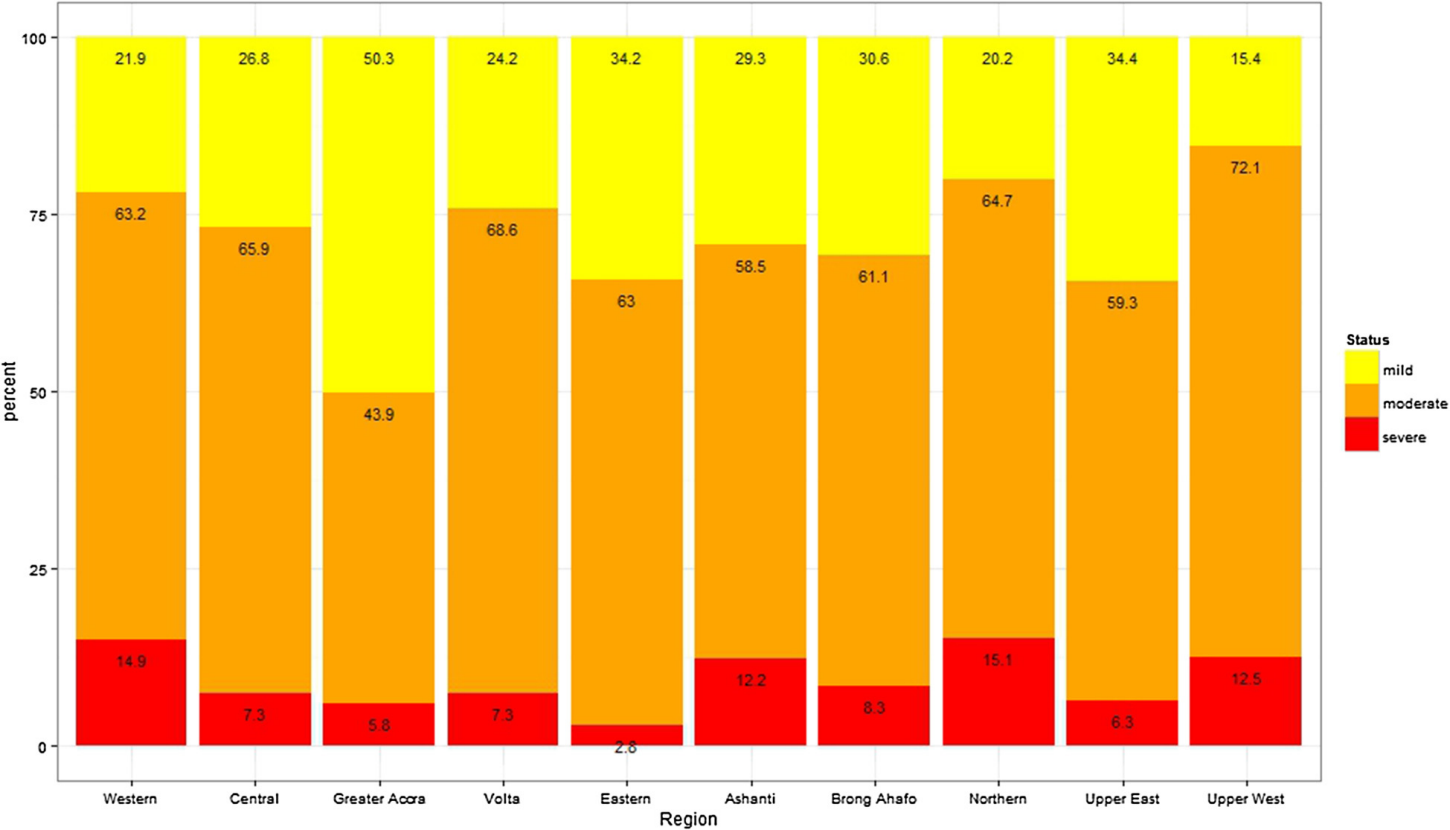
Prevalence of anemia severity across the different regions.

Of all the regions in the country, the Upper regions recorded the highest prevalence of anemia in children (Figure 1, Table 3). The results indicated that about 9 out of 10 children in the Upper East and Upper West regions were anemic, where the prevalence estimates for these two regions were 88.9% (N=158, 95% CI: 80.9-94.0) and 88.1% (N=220, 95% CI: 76.4-94.6), respectively. The lowest prevalence was recorded in the Greater Accra region, 62.3% (N=199, 95% CI: 56.0-68.3). Further investigation into the distribution of anemia among the age groups within each of the regions revealed even higher rates, where over 90% of young infants in most of the regions were anemic. Specifically, the prevalence of anemia recorded in children less than a year old in the Upper West region was as high as 97.1% (N=30, 95% CI: 52.6-100), which is over 30 percentage points higher than the overall prevalence in Africa and about 20 percentage points more than the overall national prevalence. Their counterparts in other regions such as the Northern, Western and Central regions had equally high prevalence, 91.8% (N=44, 95% CI: 78.2-97.6), 90.3% (N=21, 95% CI: 70.7-98.3), and 93.5% (N=17, 95% CI: 71.6-99.4) respectively. Approximately 98% (N=39, 95% CI: 85.3-99.9) or more of children aged 1–2 years in the Volta and Upper West regions had some form of anemia.The level of severity was also investigated across the regions (Figure 2). Most of the under-five children in all the regions had moderate levels of anemia apart from the Greater Accra region, where most of the children, 50.3% (N=157, 95% CI: 42.3-58.3) had mild anemia. In the Upper East Region, where the highest prevalence was recorded, 59.3% (N=91, 95% CI: 48.5-69.3) of the children were moderately anemic, 34.4% (N=91, 95% CI: 25.0-45.2) were mildly anemic and 6.3% (N=91, 95% CI: 2.5-14.0) had severe anemia. The Northern region recorded the highest prevalence of severe anemia, 15.1% (N=259, 95% CI: 11.1-20.2), whereas the Eastern region recorded the lowest, 2.8% (N=134, 95% CI: 0.9-7.7).

**Table 3:** Prevalence of anemia by age and region

**Region total (95% CI)**	**Characteristic age (Years)**	**Sample size (n)**	**Prevalence of anemia % (Weighted)**	**Prevalence of anemia % (Unweighted)**	p-value*
			**(95% Confidence interval)**	**(95% Confidence interval)**	
**Western**	6 months<age<1	21	90·3 (70·7-98·3)	90·5 (68·2,98·3)	0·2625
	1≤age<2	46	77·1 (64·5-89·7)	80·4 (65·6-90·1)	
	2≤age<3	46	85·3 (74·8-95·8)	89·1 (75·6-95·9)	
79·3 (73·0-84·6)	3≤age<4	38	69·1 (54·1-84·1)	73·7 (56·6,86·0)	
	4≤age<5	44	79·7 (67·6-91·8)	84·1 (69·3-92·8)	
**Central**	6 months<age<1	17	93·5 (71·6-99·4)	94·1 (69·2-99·7)	0·6521
	1≤age<2	38	84·4 (74·3-94·6)	86·8 (71·1-95·1)	
	2≤age<3	40	88·6 (79·5-97·8)	85·0 (69·5-93·8)	
85·4 (79·4-90·0)	3≤age<4	21	82·8 (68·0-97·5)	81·0 (57·4-93·7)	
	4≤age<5	30	80·5 (67·6-93·4)	80·0 (60·9-91·6)	
**Greater Accra**	6 months<age<1	14	65·4 (39·8-91·0)	64·3 (35·6-86·0)	0·0379
	1≤age<2	44	79·4 (68·4-90·4)	77·3 (61·8-88·0)	
	2≤age<3	47	55·2(42·1-68·3)	55·3 (40·2-69·5)	
62·3 (56·0-68·3)	3≤age<4	44	61·5 (48·3-74·7)	61·4 (45·5-75·3)	
	4≤age<5	50	54·1 (41·7-66·5)	52·0 (37·6-66·1)	
**Volta**	6 months<age<1	29	81·3 (66·9-95·7)	82·8 (63·5-93·5)	0·0209
	1≤age<2	39	98·0 (85·3-99·9)	97·4 (84·9-99·9)	
	2≤age<3	36	76·6 (62·0-91·3)	75·0 (57·4-87·2)	
79·5 (72·8-84·9)	3≤age<4	44	69·6 (55·6-83·7)	65·9 (50·9-79·1)	
	4≤age<5	36	73·4 (58·3-88·6)	69·4 (51·7-83·1)	
**Eastern**	6 months<age<1	22	76·9 (57·3-96·5)	77·3 (54·2-91·3)	0·5799
	1≤age<2	54	75·6 (63·5-87·8)	77·8 (64·1-87·5)	
	2≤age<3	40	81·0 (67·6-94·3)	80·0 (63·9-90·4)	
73·4 (66·3-79·5)	3≤age<4	32	67·1 (50·2-84·0)	68·8 (49·8-83·3)	
	4≤age<5	41	66·9 (51·7-82·1)	65·9 (49·3-79·4)	
**Ashanti**	6 months<age<1	32	82·7 (70·6-94·8)	84·4 (66·5-94·1)	0·3337
	1≤age<2	80	83·1 (75·7-90·4)	83·8 (73·4-90·7)	
	2≤age<3	71	81.9 (73·6-90·2)	81·7 (70·4-89·5)	
79·8 (75·5-83·5)	3≤age<4	71	80·3 (71·9-88·8)	80·3 (68·8-88·4)	
	4≤age<5	70	71·8 (62·2-81·5)	71·4 (59·2-81·3)	
**Brong Ahafo**	6 months<age<1	28	88·3 (69·2-96·8)	89·3 (70·6-97·2)	0·1683
	1≤age<2	48	85·3 (74·9-95·7)	85·4 (71·6-93·5)	
	2≤age<3	48	80·4 (68·2-92·5)	83·3 (69·2-92·0)	
78·0 (71·9-83·2)	3≤age<4	52	71·3 (59·1-83·5)	73·1 (58·7-84·0)	
	4≤age<5	42	70·1 (56·1-84·0)	71·4 (55·2-83·8)	
**Northern**	6 months<age<1	44	91·8 (78·2-97·6)	90·9 (77·4-97·0)	< 0·0001
	1≤age<2	76	92·8 (86·6-99·1)	92·1 (83·0-96·7)	
	2≤age<3	64	87·0 (78·4-95·6)	89·1 (78·2-95·1)	
82·0 (77·2-86·0)	3≤age<4	68	80·4 (70·3-90·6)	83·8 (72·5-91·3)	
	4≤age<5	83	64·8 (54·2-75·5)	68·7 (57·4-78·2)	
**Upper East**	6 months<age<1	23	86·0 (55·5-97·8)	87·0 (65·3-96·6)	0·9680
	1≤age<2	32	92·0 (70·8-98·8)	90·6 (73·8-97·5)	
	2≤age<3	28	90·9 (67·1-98·7)	85·7 (66·4-95·3)	
88·9 (80·9-94·0)	3≤age<4	32	86·2 (62·2-96·7)	84·3 (66·5-94·1)	
	4≤age<5	43	88·6 (70·0-96·8)	88·4 (74·1-95·6)	
**Upper West**	6 months<age<1	30	97·1 (52·6-100·0)	93·3 (76·5-98·8)	0·3573
	1≤age<2	64	98·7 (75·9-100·0)	98·4 (90·5-99·9)	
	2≤age<3	45	84·3 (52·7-97·3)	84·4 (69·9-93·0)	
88·1(76·4-94·6)	3≤age<4	30	78·2 (40·2-96·3)	80·0 (60·9-91·6)	
	4≤age<5	51	79·5 (49·1-94·9)	78·4 (64·3-88·2)	

*p-value shows the difference in prevalence across the various groups within each category.

**only children aged from 6 months to below 5 years are included in the analysis.

## Discussion

Results from this study showed that childhood anemia in under-five children is very high (78.4% 95% CI: 76.7-80.2) in the Ghanaian population, indicating a major health care concern. We have also observed that prevalence estimates vary across the different age and socio-demographic groups as well as the different regions and sub-regions. The highest prevalence was observed in the Upper West region, in children between the age of 1 and 2 years (98.7% prevalence), meaning almost every child between the age of 1 and 2 years, in this particular region, is affected by anemia. The prevalence for children in other age categories for this particular region was also extremely high, where at least 8 out of 10 children were found to be anemic. The lowest prevalence was observed in Greater Accra region, in children between the age of 4 and 5 years (54.2% prevalence). Severity of childhood anemia also varied among the different sub-groups of the population.

The high prevalence among infants less than 2 years of age in the overall population and in particular among the rural children is of special concern. For infants under 2 years, this would likely be due to: a) high prevalence of maternal micronutrient deficiency since children born to malnourished mothers have poor stores of iron, zinc, vitamin A and B_12_ and folate [19-21], b) low concentration of iron in breast milk which may be insufficient to meet the daily iron requirements of the infant [3] c) the introduction of complementary foods often occurs within this period which is also a period for rapid physical development with increased blood volume and a decrease in iron storage from maternal source [21] d) the susceptibility of infants to infections and diseases, which affects their nutrition and feeding and thus decreases the ability of their body to ingest and absorb iron [22].

The differences in the prevalence of anemia between younger children less than 2 years old and older ones aged 2–5 years were statistically significant. The decline in prevalence among the older children supports similar findings in several previously reported literatures [3,4,12,23]. One main reason is the decrease in iron requirements and increase in iron intakes with age [12]. In Ghana, mostly in rural areas, beef, eggs and other kinds of heme-containing foods (meat) are only introduced to the diet of children after weaning, which is often after 18–24 months [24]. Moreover, unlike the younger children, the older children are less susceptible to infections and diseases which inhibit their iron absorption [25].

The high prevalence rate of anemia in the rural part of the country can also be attributed to: a) malnutrition due to limited consumption of foods rich in micronutrient as a result of poverty and less favourable socio-economic status b) lack of good drinking water and better sanitation facilities [23], leading to higher rates of infections and diseases and consequently increased risk of anemia. The results from the analysis actually confirm this hypothesis, since the prevalence among children who had infections was significantly higher than among children with none of the 3 infections: diarrhea, fever, and cough. c) Limited access to better and improved health care systems.

The high incidence of anemia in the country especially in the Northern parts as well as the Central region can be linked to the fact that most of the areas in these regions are rural (80% and above) with higher poverty rates and lower levels of education [13]. Furthermore, Ghana is a malaria-endemic country with intense malaria transmission in the Northern and Upper regions [9]. Since Plasmodium falciparum is known to be one of the leading etiological factors of childhood anemia, the results of the study is consistent with the literature [25]. In the same vein, the low prevalence rate observed in the Greater Accra region could be due to the high proportion of urban areas in the region, 88.5%. However, it was interesting to find out that despite the fact that the proportion of urban areas in the Eastern is only about 32% compared to the Ashanti region with about 40% urban areas, the prevalence rate was higher in the Ashanti region than in the Eastern region.

Following the 2008 GDHS report, efforts have been made by Ministry of Health, the Ghana Health Service, Food and Drugs Board and other government and non-governmental organizations to help improve the nutrition of especially children and pregnant women over the years [26,27]. The interventions that have been implemented include promotion of exclusive breastfeeding for the first 6 months of life, promotion of appropriate complementary feeding at 6 months with continued breastfeeding until 2 years and vitamin A and iron supplementation for children aged 6 months to 5 years, which was implemented in some parts of the country. However, the impact of these interventions has yet to be seen in future studies and reports.

It is important to note that in addition to the GDHS, Ghana also conducts a multiple indicator cluster survey (MICS). The report from the latest (2011) survey indicates that the population level prevalence of childhood anemia for under-five children is 57% [28], which is much lower than what is observed in our analysis and in the 2008 GDHS report, which is very promising. This might have been due to the interventions. However, further in depth analysis of the MICS data set and the latest (2013) GDHS data set (which is currently underway) is required to examine, if the decrease prevalence of anemia is also achieved in the most affected socio-demographic groups and regions.

Finally, we would like to note that our population estimates are consistent with the 2008 GDHS report. However, our prevalence estimates for male and female children (79.6% and 77.1%, respectively) were slightly different from those provided in the 2008 GDHs report (79.1% and 76.6%, respectively). Also, our estimated urban and rural prevalence (67.5% and 84.8%) were also slightly different from the 67.9% and 84.1% reported in the 2008 GDHS report. We have investigated this further and observed that the number of children, in these sub-groups, in the GDHS report is different from what we have in our analysis. However, we were not able to explain this discrepancy in sample size.

## Conclusion

This study has accentuated evidence of an extremely high prevalence of childhood anemia in Ghana, especially in certain sub-groups of the population. Based on the WHO criteria, a population prevalence rate of greater than 40% is a severe public health concern requiring immediate actions and measures to help reduce the burden of the disease. Specific groups with urgent needs include children aged 6 months to 2 years, children in the rural areas and children in the Northern and Upper regions as well as the Central region. Further research, by comparing subgroups of the population with high and low prevalence, is required to determine potential risk factors associated with the prevalence of childhood anemia. Further study is also needed to determine appropriate targeted interventions.

## Competing interests

The authors declare that they have no competing interests.

## Authors’ contributions

JEE participated in the conception of the study, performed the statistical analysis and drafted the first version of the manuscript. CA acquired the data and participated in the critical review of the manuscript. JB participated in the critical review of the manuscript. JSH participated in the conception of the study, provided methodological guidance, supervised the analysis and participated in drafting the manuscript. All authors read and approved the final manuscript.

## Pre-publication history

The pre-publication history for this paper can be accessed here:

http://www.biomedcentral.com/1471-2458/14/626/prepub
